# Distinct Immunophenotypic Features in Patients Affected by 22q11.2 Deletion Syndrome with Immune Dysregulation and Infectious Phenotype

**DOI:** 10.3390/jcm12247579

**Published:** 2023-12-08

**Authors:** Giorgio Costagliola, Annalisa Legitimo, Veronica Bertini, Antonino Maria Quintilio Alberio, Angelo Valetto, Rita Consolini

**Affiliations:** 1 Section of Pediatric Hematology and Oncology, Azienda Ospedaliero-Universitaria Pisana, 56126 Pisa, Italy; giorgio.costagliola@hotmail.com; 2 Section of Clinical and Laboratory Immunology, Pediatric Unit, Department of Clinical and Experimental Medicine, University of Pisa, 56126 Pisa, Italy; a.legitimo@ao-pisa.toscana.it; 3 Section of Cytogenetics, Department of Laboratory Medicine, Azienda Ospedaliero-Universitaria Pisana, 56126 Pisa, Italy; v.bertini@ao-pisa.toscana.it (V.B.); a.valetto@ao-pisa.toscana.it (A.V.); 4 Pediatrics Unit, Department of Clinical and Experimental Medicine, University of Pisa, 56126 Pisa, Italy; antonino.alberio@gmail.com

**Keywords:** autoimmunity, dendritic cells, immune dysregulation, infections, memory T cells, naïve T cells, recent thymic emigrants

## Abstract

The clinical expression of 22q11.2 deletion syndrome (22q11.2 DS) is extremely variable, as patients can present with recurrent or severe infections, immune dysregulation, atopic diseases, or extra-immunological manifestations. The immunological background underlying the different disease manifestations is not completely elucidated. The aim of this study was to identify the immunophenotypic peculiarities of 22q11.2 DS patients presenting with different disease expressions. This study included 34 patients with 22q11.2 DS, divided into three groups according to the clinical phenotype: isolated extra-immunological manifestations (G1), infectious phenotype with increased/severe infections (G2), and immune dysregulation (G3). The patients underwent extended immunophenotyping of the T and B lymphocytes and analysis of the circulating dendritic cells (DCs). In patients with an infectious phenotype, a significant reduction in CD3+ and CD4+ cells and an expansion of CD8 naïve cells was evidenced. On the other hand, the immunophenotype of the patients with immune dysregulation showed a skewing toward memory T cell populations, and reduced levels of recent thymic emigrants (RTEs), while the highest levels of RTEs were detected in the patients with isolated extra-immunological manifestations. This study integrates the current literature, contributing to elucidating the variability in the immune status of patients with 22q11.2DS with different phenotypic expressions, particularly in those with infectious phenotype and immune dysregulation.

## 1. Introduction

22q11.2 deletion syndrome (22q11.2 DS) is a multisystemic embryopathy featuring a wide spectrum of manifestations and significant clinical and immunological heterogeneity. It is caused by a hemizygous microdeletion on the q arm of chromosome 22, at the 11.2 locus, and its pathogenesis relies on a developmental defect in the third pharyngeal pouch and fourth pharyngeal arch that causes abnormalities in the parathyroid glands, thymus, heart, face, and palate [1]. The genomic region affected contains a considerable number of genes with different functions, including the TBX1 gene, which is pivotal for thymic development. Notably, the expression of TBX1, as well as the clinical phenotype, is influenced by a wide number of genetic modifiers, thus contributing to the disease heterogeneity of 22q11.2DS [2]. Immune deficiency is secondary to thymic hypoplasia and has a variable spectrum, ranging from a T-negative severe combined immunodeficiency (SCID) to a mild T cell defect [3]. Consequently, patients have a higher risk for recurrent or severe infections, and also immune dysregulation and atopic diseases are frequently observed. On the other hand, some patients display only extra-immunological manifestations, including congenital heart defects, hypoparathyroidism, neuropsychiatric disorders, velo-pharyngeal dysfunction, and others [4]. 

In order to identify patients early with 22q11.2 DS at an increased risk of these heterogeneous manifestations, different attempts to provide a correlation between the immunophenotypic and clinical features have been performed, mainly focusing on patients with autoimmunity or allergy. However, despite some promising results, previous studies have been markedly heterogeneous concerning patient selection, categorization, and immunophenotypic approach [5,6,7]. Therefore, this intriguing aspect is far from being fully explored. In this study, we performed a retrospective analysis on patients with 22q11.2 DS with different clinical expressions, to identify the immunophenotypic peculiarities of those presenting with an infectious phenotype or immune dysregulation. 

## 2. Materials and Methods

### 2.1. Patient Selection

This study is a retrospective analysis that includes 34 patients with 22q11.2 DS diagnosed and followed at the Immunology Department of the University of Pisa. The diagnosis of 22q11.2DS was performed through the demonstration of the chromosome 22q11.2 deletion by multicolor fluorescent in situ hybridization (FISH) or comparative genomic hybridization (CGH) array. Only patients with at least 12 months of follow-up were included in this study, to allow for a correct clinical characterization of the analyzed cohort. All the patients of this cohort are included in the Italian Primary Immunodeficiency Network (IPINet) 22q11DS National Registry. Patients with a 22q11.1DS-like phenotype without the evidence of the chromosome 22q11.2 deletion were excluded, as well as patients receiving immunosuppressive agents at the time of sampling.

### 2.2. Clinical Data Collection

For each patient, demographic and clinical data were retrospectively collected, including the age at diagnosis, and the clinical manifestations, especially in terms of infectious burden and presence of signs of immune dysregulation (autoimmunity, lymphoproliferation, atopy, enteropathy). 

### 2.3. Definition of the Study Subgroups

Based on their clinical expression, we divided the patients into 3 groups: patients with isolated extra-immunological manifestations (G1), infectious phenotype with increased/severe infections (G2), and immune dysregulation (G3). Patients were considered as having an infectious phenotype (G2) in case of a history of severe infections requiring hospitalization or parenteral antibiotic therapy or when the frequency of infections was above the normal limit for that age. Specifically, concerning respiratory infections, the cut-off used was the presence of 6 or more infections per year in children aged 1–3 years, 5 or more infections per year for those aged 3–6 years, and 3 or more infections per year for patients with age greater than 6 years. The cut-off values were chosen according to the definition of recurrent respiratory infections adopted by the Italian Society for Paediatrics [8]. On the other hand, recurrent urinary tract infections were defined as at least 3 episodes per year or at least 2 episodes in 6 months [9].

Immune dysregulation was defined by the presence of autoimmune diseases, lymphoproliferation, severe atopic disease, or enteropathy. Autoimmune diseases were confirmed with clinical or laboratory criteria, and lymphoproliferation was defined by the presence of chronic or recurrent lymphadenopathy, splenomegaly, or lymphocytic tissue/organ infiltration, while enteropathy was defined according to clinical and endoscopic features. Severe atopic disease was defined according to the specific scores used in clinical practice for the different conditions (i.e., SCORing Atopic Dermatitis [SCORAD] for atopic dermatitis) [10]. Mild allergic diseases (i.e., allergic rhinitis) were not considered in the definition of subgroups. All the patients presenting with features of immune dysregulation were classified into G3, independently from the infectious pattern.

### 2.4. Flow Cytometric Analysis

For each patient, eight-color flow extended immunophenotyping of T and B lymphocytes and the analysis of circulating dendritic cells (DCs) were performed according to standard protocols; blood was collected in vacutainer tubes containing ethylene-diamine-tetra-acetic acid (EDTA) as anticoagulant and processed within 5 h of collection. Details of the panels, specification of antigens, and gating strategies are provided in the Supplementary Materials (Table S1, Figures S1 and S2). Briefly, 100 µL of peripheral blood were stained with surface antibodies against CD3, CD4, CD8, CD19, and CD16/56 antigens. CD3+CD4+ (helper) and CD3+CD8+ (cytotoxic) lymphocytes were also analyzed for expression of CD45RA, CD62L, and CD31 to identify naïve (CD45RA+CD62L+), central memory (CD45RA−CD62L+), effector memory (CD45RA−CD62L−), terminal effector cells (CD45RA+CD62L−), and recent thymic emigrants (RTE, CD45RA+CD62L+CD31+). Circulating regulatory T cells (Treg) were identified as a CD4+CD25+/++CD127low/− cell population. The expression of CD45RA was evaluated to estimate the amount of naïve Treg cells. The expression of CD185 (CXCR5) on CD3+CD4+CD45RO+ T lymphocytes was investigated in order to identify T follicular helper (TfH) cells. The following B lymphocyte subpopulations were also determined: naive (CD19+CD27−IgD+), switched memory (CD19+CD27+IgM−IgD−), transitional (CD19+CD38++IgM++), and CD21low (CD19+CD21lowCD38low). Circulating DCs were phenotypically characterized directly into myeloid dendritic cells (mDCs) and plasmacytoid dendritic cells (pDCs). To identify DCs, cells were stained with the following antibodies: CD14+CD16, CD85k, CD33, or CD123 for the mDC and pDC subsets, respectively. Dendritic cells were identified as CD14low/−CD16low/−CD85k+ and CD33+ or CD123+, as previously reported by our group [11]. The samplings were performed after the exclusion of acute infections. At the time of sampling, patients had not received any immunosuppressive therapy (including systemic corticosteroids) for at least two months. 

### 2.5. Statistical Analysis

Data are expressed as mean and standard deviations. The comparisons among the 3 subgroups were performed through ANOVA analysis and subsequent t-tests among the single groups. Data are graphically presented with box plots, in which all the single values are displayed. All the analyses were performed with the GraphPad Prism software (version 10.1.0). In this study, a *p*-value <0.05 was considered to be statistically significant.

## 3. Results

### 3.1. Demographic and Clinical Features

Thirteen patients were included in G1, 10 in G2, and 11 in G3, respectively. The demographic data of the included patients are summarized in Table 1. The patient’s age was found not to be statistically different among the three groups (*p* = 0.2606), as well as the frequency of the other clinical manifestations. The most commonly observed extra-immunological manifestations consisted of neuropsychiatric involvement (23/34) and defects in phospo-calcium homeostasis (17/34). One patient of G3 had localized papillary thyroid carcinoma, which was treated with surgery. Lymphocyte phenotyping was performed after the diagnosis of malignancy.

Immune dysregulation in G3 consisted of thyroid disease (*n* = 5), immune thrombocytopenia (ITP) (*n* = 3), cutaneous autoimmunity (psoriasis/alopecia, *n* = 3), undifferentiated connective tissue disease (*n* = 1), and severe eczema (*n* = 1). In the present cohort, there were no cases of lymphoproliferation or enteropathy. One patient of G3 developed thyroid cancer during the follow-up. All the patients included in the present cohort are alive. 

### 3.2. Patients with Infectious Phenotype Have Low CD3+ and CD4+ Cells and Elevated CD19+ Cells

Differences among the three groups were evidenced in the conventional lymphocyte subpopulations, extended lymphocyte phenotyping, and DC values (detailed results in Table 2).

The absolute number of lymphocytes was not significantly different between the subgroups, although the patients with immune dysregulation showed the lowest values, especially compared with the patients with an infectious phenotype (G2: 2690 ± 1080; G3: 1950 ± 650; *p*-value: 0.1025). The percentage values of the CD3+ cells were found to be significantly lower in the patients presenting with an infectious phenotype compared to those with immune dysregulation (G2: 56.42 ± 10.84; G3: 67.96 ± 9.61; *p*-value < 0.01), although this difference was not significant in the absolute values. Also, the percentage of CD4+ cells was significantly lower in the patients with an infectious phenotype in comparison with those with immune dysregulation (G2: 32.91 ± 6.19, G3: 43.53 ± 8.80; *p*-value < 0.05).

**Figure 1:**
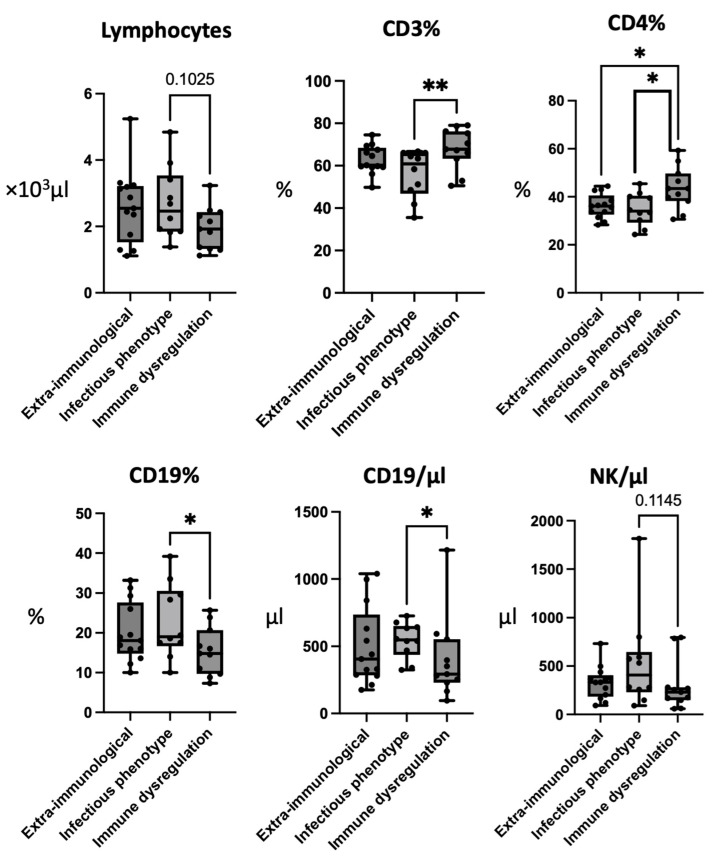
Comparison of conventional lymphocyte subpopulations between 22q11.2 patients with extra-immunological phenotype (G1), infectious phenotype (G2), and immune dysregulation (G3). Figure legend: * is used to indicate *p*-values <0.05; ** is used for *p*-values <0.01.

On the other hand, the percentage of CD19+ cells was significantly elevated in the patients with an infectious phenotype compared with those with immune dysregulation (G2: 22.77 ± 9.35; G3: 15.37 ± 6.09; *p*-value < 0.05). This difference was also evidenced in the absolute values of the CD19+ cells (G2: 545.2 ± 135.36; G3: 402.73 ± 308.26; *p*-value < 0.05). Moreover, the patients with immune dysregulation showed the lowest absolute levels of the CD19+ and NK cells (Figure 1).

### 3.3. Patients with Immune Dysregulation Have Reduced Recent Thymic Emigrants

The analysis of RTEs showed the highest levels were in the patients presenting with isolated extra-immunological involvement. The difference with the group of patients with immune dysregulation, which showed the lowest levels of RTEs, was statistically significant (G1: 43.45 ± 18.06; G3: 25.39 ± 10.20; *p*-value < 0.05).

### 3.4. Patients with Immune Dysregulation Have Higher CD4+ Memory Cells

Concerning the CD4+ population, a skewing toward memory subsets was observed in the patients with immune dysregulation, who showed the highest percentage levels of CD4+ effector memory (EM) and central memory (CM) cells compared with the other groups. The difference in the levels of the CD4+ CM cells between the patients with immune dysregulation and those with extra-immunological phenotype was statistically significant (G1: 22.38 ± 14.78; G3: 34.3 ± 10.35; *p*-value < 0.05), while the comparison between the values of the CD4+ EM cells did not reach statistical significance (G1: 14.02 ± 10.27; G3: 24.18 ± 14.59; *p*-value: 0.0548).

### 3.5. Patients with Immune Dysregulation Have Higher CD8+ Memory Cells and Patients with Infectious Phenotype Have High CD8+ Naïve Cells

**Figure 2:**
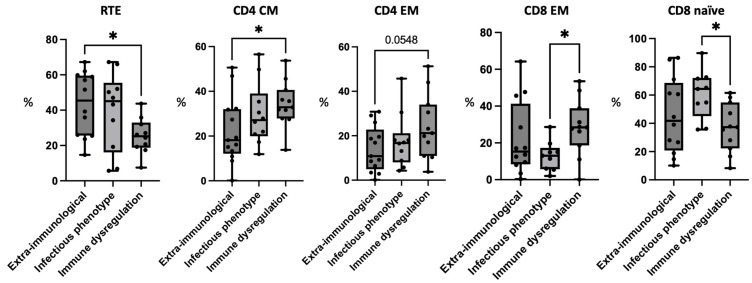
Comparison of T cell subpopulations between 22q11.2 patients with extra-immunological phenotype (G1), infectious phenotype (G2), and immune dysregulation (G3). Figure legend: * is used to indicate *p*-values <0.05; CM: central memory; EM: effector memory; RTE: recent thymic emigrants.

The prevalence of memory cells in the patients with immune dysregulation was observed also in the CD8+ compartment, with the lowest values detected in the patients with an infectious phenotype. The difference between these two groups was statistically significant concerning the CD8+ EM population (G2: 12.88 ± 8.1; G3: 28.43 ± 15.35; *p*-value < 0.05). On the other hand, the patients with infections showed the highest levels of CD8+ naïve cells, which were significantly elevated compared to the group with immune dysregulation (G2: 60.45 ± 17.48; G3: 37.05 ± 17.37, *p*-value < 0.05). The data for the main differences in the T cell extended phenotyping are summarized in Figure 2.

### 3.6. Regulatory T Cells, Follicular T Cells, and B Cell Phenotyping

The values of the regulatory T cells (Tregs) and follicular T cells did not show significant differences among the three groups. The analysis of the B cell subsets did not show significant differences in the analyzed groups.

### 3.7. Patients with an Infectious Phenotype Have Higher Levels of Peripheral Dendritic Cells

**Figure 3:**
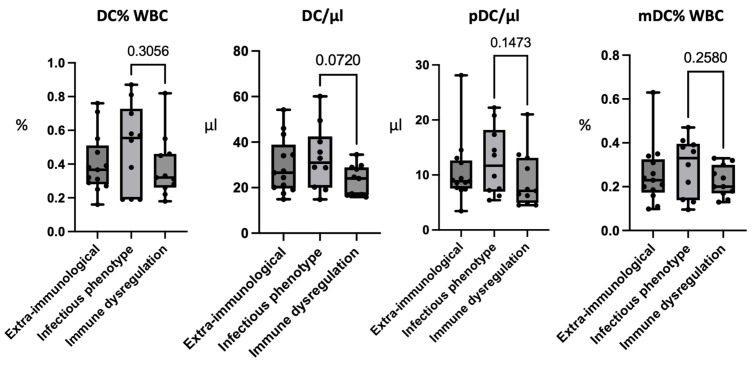
Comparison of dendritic cells populations between 22q11.2 patients with extra-immunological phenotype (G1), infectious phenotype (G2), and immune dysregulation (G3). Figure legend: DCs: dendritic cells; mDCs: myeloid dendritic cells; pDCs: plasmacytoid dendritic cells.

The analysis of the DCs, although not providing statistically significant results, showed a tendency to present elevated percentage values of DCs in the patients with infectious phenotype, especially compared to those presenting with immune dysregulation, as summarized in Figure 3 (G2 0.50 ± 0.26; G3 0.38 ± 0.18; *p*-value: 0.3056). A difference between the two groups was observed also in the absolute values of the DCs (G2: 33.02 ± 14.01; G3: 23.05 ± 6.63; *p*-value: 0.072). Concerning the analysis of the DC subsets, the patients with an infectious phenotype had the highest absolute values of pDCs, with the lowest values being observed in the patients with immune dysregulation (G2: 12.46 ± 6.17; G3: 9.11 ± 5.14; *p*-value: 0.1473). In this group, higher percentage values of mDCs were also observed, especially in comparison with the patients with immune dysregulation (G2: 0.29 ± 0.13; G3: 0.22 ± 0.07; *p*-value: 0.258).

## 4. Discussion

22q11.2DS is a heterogeneous disease, and its genetic and immunologic backgrounds are complex, with the knowledge of these aspects being in continuous evolution. The immune defect responsible for the increased susceptibility to recurrent and severe infection relies not only on the degree of thymic hypoplasia or complete aplasia, but also on recently described factors, including an altered thymic organization (with impaired cortical/medullary differentiation) and a perturbed distribution of thymocytes [12]. Based on these variables, 22q11.2DS can present with different degrees of T cell deficiency, ranging from patients with a normal or slightly reduced T cell number and function to severe conditions featuring the complete absence of T cells, thus presenting with a SCID-like clinical and immunological phenotype [12]. In patients with 22q11.2DS, the most commonly reported immunological findings are represented by reduced T cell levels with decreased thymic output, which is expressed by low levels of RTEs and naïve T CD4+ and CD8+ cells. Also, a restricted T cell receptor (TCR) repertoire is described in the disease [13]. However, given the impact of T cells in immune homeostasis, an impairment of the B cell response has also been reported in patients with 22q11.2DS, and patients can show hypogammaglobulinemia or defective B cell maturation, expressed by reduced levels of switched memory B cells and the lack of an adequate humoral response against vaccination [14,15]. Notably, B cell impairment and hypogammaglobulinemia usually became more evident with the increase in age, while in childhood, the most common aspect is a reduction in somatic hypermutation [15]. T cell defects and, when associated, B cell alterations, are finally responsible for the infectious phenotype observed in the disease, which is expressed mainly with recurrent infections, such as prolonged viral infections and frequent superinfections of the upper and lower respiratory tracts. The infectious susceptibility is also influenced by the congenital anomalies associated with 22q11.2DS, including the altered anatomical structure and functional impairment of the pharynx and ear, thus complicating the approach to this challenging category of patients [2,3]. 

The immune defect is not only responsible for the infectious phenotype. Indeed, patients with 22q11.2DS have an impairment of the central immunological tolerance, with an ineffective negative selection of thymocytes, which partly depends on an abnormal thymic environment with the defective expression of AIRE [12,16]. Patients also show reduced production of Tregs, with a tendency towards activated Treg cell phenotypes and low levels of naïve Tregs, finally resulting in the expansion of self-reactive T cells and an increased susceptibility to immune dysregulation [7,17,18]. Notably, the reduction in Tregs is typically more evident with the increasing of age, and this partially reflects the temporary development of autoimmune manifestations in 22q11.2DS [3,16]. Additionally, the ineffective immune response causes the persistence of microbial antigens, thus potentially favoring the phenomenon of molecular mimicry and eliciting autoimmunity [3,19]. 

In recent years, there has been an increasing interest in the features of immune dysregulation in patients with inborn errors of immunity (IEI), including 22q11.2DS. Indeed, it has been demonstrated that about 10% of the patients with IEI present needing clinical attention for the presence of the isolated signs of immune dysregulation, which are reported in a considerable fraction of patients during the disease course [20]. The most commonly observed signs of immune dysregulation in IEI are represented by autoimmune cytopenia, which have been found with a 120-fold higher frequency in patients with IEI compared to the general population [21]. Other frequent dysregulatory features are autoimmune endocrinopathies (mostly thyroiditis), rheumatologic manifestations, increased susceptibility to atopic diseases, and lymphoproliferation [16]. In this wider clinical context, the problem of immune dysregulation is pivotal also in 22q11.2DS, as recent studies have demonstrated that autoimmune manifestations are diagnosed in up to 10% of patients with 22q11.2DS [16,22,23], while the prevalence of clinically relevant allergic diseases is less defined. In 22q11.2DS, the most commonly described autoimmune diseases are cytopenia, thyroiditis, and arthritis, with a markedly increased incidence compared with the general population [16]. Given the partly unexplored immunological background of the disease and the clinical and immunophenotypic variability of 22q11.2DS, the search for immunological features associated with increased risk for immune dysregulation is of extreme clinical relevance, as well as the identification of patients with higher risk for severe and life-threatening infections.

This study, by comparing the immunophenotypic features of patients with 22q11.2DS and different clinical expressions, evidenced some peculiarities, with potential relevance for clinical practice, that were especially identified in patients with an infectious phenotype and those with immune dysregulation. Specifically, patients with an infectious phenotype showed significantly reduced CD3+ and CD4+ cells and an expansion of CD8 naïve cells, while those with immune dysregulation displayed a skewing toward memory T cell populations, with reduced levels of RTEs, and low levels of CD19 B cells. 

Comparing our data with the currently available literature, it emerged that reduced CD19 cells in patients with autoimmunity have already been reported in a study by Desphande et al. in 2020 [24], in association with CD3+ lymphopenia. Low levels of CD3+ cells in patients with autoimmunity have also been evidenced in a recent large cohort study by Crowdley et al., which also demonstrated reduced levels of CD4+ cells in patients with autoimmune thyroid disease and reduced CD4 naïve cells in those with ITP [25]. Notably, in both of the studies, the infectious phenotype was not considered in the categorization of the patients. In this paper, the inclusion of a separate subgroup composed of patients with increased or severe infections allowed us to demonstrate, as expected, a more profound relative T cell defect in this population. Indeed, CD3 levels < 50% have previously been associated with a higher risk of immune dysregulation [7], but thresholds associated with an increased risk of infections are lacking. The extension of the analysis to a larger cohort of patients could identify specific thresholds useful for routinely clinical practice, and especially for the identification of patients who would benefit from antimicrobial prophylaxis. Similarly, defining thresholds associated with an increased risk of immune dysregulation could help in providing an appropriate follow-up with screening for autoimmune, atopic, and lymphoproliferative diseases.

**Figure 4:**
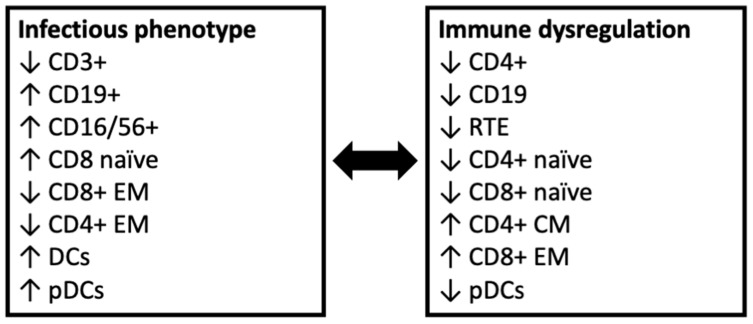
Proposed immunophenotype-based definition of subgroups of 22q11.2 patients with increased risk for infections or immune dysregulation. Figure legend: The figure summarizes the main immunophenotypic features evidenced in the subgroups of patients with recurrent or severe infections (infectious phenotype) and immune dysregulation. CM: central memory; DCs: dendritic cells; EM: effector memory; pDCs: plasmacytoid dendritic cells; RTE: recent thymic emigrants.

Concerning the extended lymphocyte phenotyping, the findings from this study are in line with data deriving from a study by Montin et al., in which patients affected by ITP and/or autoimmune hemolytic anemia showed reduced levels of CD4+ naïve cells, RTEs, and switched memory B cells compared to patients without hematologic autoimmunity [26]. Similarly, a large monocentric cohort published by Giardino et al. showed T cell lymphopenia and a skewing towards CD4+ memory cells, with consequently reduced naïve CD4+ cells, in patients with autoimmunity [27]. In the present study, the skewing towards memory populations was also evidenced in CD8+ cells, contributing to identifying another feature that points out the profound differences in the immune phenotype of patients with immune dysregulation. Moreover, in our cohort, the definition of a specific subgroup of patients with isolated extra-immunological manifestations allowed us to demonstrate, as expected, that patients who do not present with relevant infections or immune dysregulation have a less profound thymic impairment, showing the highest levels of RTEs. Although, with the limitations deriving from the small sample size, the retrospective nature of this study and the differences in the age range of the subgroups, data deriving from the present study, together with the knowledge available from current literature, suggest the possibility of providing an immunophenotype-oriented classification of 22q11.2DS patients, as is provisionally proposed in Figure 4. Due to the mentioned limitations, the results deriving from this study are not conclusive, and further studies on larger cohorts and with a more homogeneous age range are needed to confirm our findings and this provisional categorization. Moreover, a better characterization of the genetic background of the disease and its genetic modifiers could potentially help in providing a more personalized approach to patients with 22q11.2DS [2].

Additionally, this is the first study evidencing differences in DCs according to the disease manifestations. It is well-known that DCs can be classified into different subsets, each with a different function in the response to pathogens and immune homeostasis, thus being an important factor in enhancing the ability of the immune system to differentiate the immune response [28]. As a perturbation in the DC number, function, and differentiation could be responsible for an altered response against pathogens and immune dysregulation, an analysis of these factors in patients with IEI could help in dissecting the clinical heterogeneity of the diseases, and specifically to improve the knowledge of the basis of the development of immune dysregulation. In the variability of DCs, the two main populations are represented by mDCs and pDCs, which show significant differences in their transcriptional program, development, and immunological functions [28,29], although both populations are implicated in stimulating the T-helper immune response and promoting the initiation of not only the immune response against pathogens but also immune tolerance [28,29]. Specifically, mDCs have a strong immunoregulatory phenotype, as they are able to produce interleukin-10 (IL-10) and soluble CD25 after stimulation with pathogens [30,31]. On the other hand, pDCs have a relevant role in the immune response against infections, as they produce high levels of interferon (IFN)-α, which has marked antiviral properties. Moreover, pDCs support B cell function and development, thus being a co-factor in the humoral response against pathogens [32] and, interestingly, also act by promoting mDC differentiation and mDC-mediated T cell responses [33,34] in a complex immunological crosstalk. Previously, we demonstrated that the circulating levels of DCs are reduced in patients with 22q11.2DS compared with healthy controls [35], but a correlation with the clinical phenotype of the disease is still lacking. The current study, although not reaching statistical significance, evidenced a tendency towards a reduction in DCs in patients with immune dysregulation. Given the known role of mDCs in promoting immune tolerance and pDCs in supporting B cell function and antiviral response, the different DC expression represents an intriguing aspect that needs to be investigated in further studies on larger cohorts of patients, to better define the role of this cell population in the clinical heterogeneity of 22q11.2DS. 

## 5. Conclusions

Our findings integrate the current literature and contribute to elucidating the variability in the immune status of patients with 22q11.2DS showing different phenotypic expression, particularly in those with infectious phenotype and immune dysregulation. These differences could potentially be helpful for routinely clinical practice, helping in the identification of patients who would benefit from antibiotic prophylaxis or who should be screened for underlying immune dysregulation. Further investigations, performed on larger cohorts, are needed to confirm and integrate the evidence deriving from this study. Hopefully, the adoption of more homogeneous strategies for extended immunophenotyping and for the definition of subgroups for clinical studies could significantly improve the knowledge of this intriguing aspect of 22q11.2DS. 

## Figures and Tables

**Table 1:** Clinical and demographic features of the included patients.

	G1	G2	G3
Age (years)	13.61 ± 10.71	10.88 ± 9.31	18.35 ± 11.26
Congenital heart disease requiring cardiosurgery	3/13	3/10	4/11
Hypoparathyroidism/hypocalcemia	4/13	6/10	7/11
Neuropsychiatric involvement	8/13	8/10	7/11
Velo-pharyngeal insufficiency or ear/nose abnormalities	4/13	3/10	2/11
Other relevant malformations (kidney, gastrointestinal, limbs)	3/13	3/10	4/11
Allergic disorders	2/13	2/10	2/11
Neoplasia	0/13	0/10	1/11

**Table 2:** Detailed results of the relative and absolute numbers of lymphocyte subsets and peripheral dendritic cells.

		G1	G2	G3
Lymphocytes	/μL	2600 ± 1120 (1110–5240)	2690 ± 1080 (1380–4840)	1950 ± 650 (1120–3230)
CD3	%	62.81 ± 6.67 (49.8–74.5)	56.42 ± 10.84 (41.8–66.7)	67.96 ± 9.61 (50.5–78.7)
	/μL	1634.0 ± 717.52 (753–3102)	1552.3 ± 708.96 (577–2608)	1323.82 ± 512.67 (753–2552)
CD4	%	36.16 ± 5.04 (28.3–44.4)	32.91 ± 6.19 (24.3–41.5)	43.53 ± 8.80 (32–59.3)
	/μL	954.01 ± 463.17 (326–1907)	923.4 ± 474.49 (335–1621)	851.0 ± 394.76 (486–1915)
CD8	%	18.58 ± 4.84 (10.2–27)	15.91 ± 5.29 (11–24.7)	20.17 ± 5.66 (14.3–32.6)
	/μL	455.0 ± 211.32 (167–894)	436.6 ± 232.72 (152–767)	390.36 ± 163.62 (205–753)
CD19	%	20.07 ± 7.49 (10–33.2)	22.77 ± 9.35 (10–39.2)	15.37± 6.09 (7.3–25.7)
	/μL	500.77 ± 292.66 (175–998)	545.2 ± 135.36 (341–727)	402.73 ± 308.26 (96–1215)
CD16/56	%	13.98 ± 6.86 (5.2–30)	18.06 ± 10.62 (6.6–37.5)	15.41 ± 11.25 (2.5–32.3)
	/μL	329.69 ± 171.29 (92–732)	537.2 ± 501.97 (91–1815)	293.36 ± 256.92 (59–796)
CD4				
RTEs ^a^		43.45 ± 18.06 (14.7–67.2)	39.16 ± 22.26 (5.7–67.2)	25.39 ± 10.20 (7.5–43.7)
Naïve ^a^		53.18 ± 25.3(20–87.6)	50.21 ± 25.03 (10–80.5)	38.45 ± 18.94 (12–69.5)
TEMRA ^a^		10.4 ± 11.97(0.3–36.2)	2.11 ± 2.19 (0.3–7.3)	3.05 ± 4.62 (0.4–16)
CM ^a^		22.38 ± 14.78 (0.1–50.6)	29.84 ± 14.06 (11.9–56.5)	34.3 ± 10.35 (13.8–53.7)
EM ^a^		14.02 ± 10.27 (0.1–30.8)	17.76 ± 12.36 (5.7–45.7)	24.18 ± 14.59 (3.8–51.3)
CD8				
Naïve ^b^		45.51 ± 26.96 (10.1–86.4)	60.45 ± 17.48 (35.6–89.7)	37.05 ± 17.37 (8.3–61.5)
TEMRA ^b^		19.69 ± 15.4 (5.1–56.5)	14.39 ± 7.08 (6.7–30.3)	19.6 ± 15.48 (0.1–54)
CM ^b^		9.98 ± 7.73 (0.9–26.7)	12.27 ± 8.78 (1.2–28.5)	16.5 ± 10.02 (1.5–38.3)
EM ^b^		22.28 ± 20.02 (0.3–64.2)	12.88 ± 8.1 (2–28.6)	28.43 ± 15.35 (0.1–53.5)
Tregs ^a^		7.04 ± 2.69 (4.3–11.4)	7.55 ± 1.69 (4.9–15)	6.31 ± 3.21 (2.8–10.3)
TfH ^c^		31.51 ± 8.1 (19.5–42.4)	33.36 ± 6.89 (23.8–45.7)	26.81 ± 5.57 (19.1–35.4)
CD19				
Naïve ^d^		79.91± 7.42 (66.6–92)	78.32 ± 5.67 (69.5–88.2)	76.74 ± 9.07 (60.1–88.2)
Switched memory ^d^		6.64 ± 4.74 (2.3–15)	7.58 ± 4.0 (1.1–14)	6.05 ± 3.39 (2.5–13.74)
Transitional ^d^		0.92 ± 0.57 (0.3–1.9)	0.72 ± 0.75 (0.05–2.17)	2.07 ± 3.76 (0.1–9.7)
CD21low ^d^		6.16 ± 2.75 (3.4–10.3)	8.04 ± 3.81 (3.4–14.2)	5.71 ± 2.73 (2.3–10.5)
DC ^e^		0.4 ± 0.18 (0.27–0.76)	0.50 ± 0.26 (0.19–0.87)	0.38 ± 0.18 (0.18–0.82)
DC	/μL	29.45 ± 12.2 (14.9–54.2)	33.02 ± 14.01 (14.8–60.1)	23.05 ± 6.63 (15.89–34.5)
pDC ^e^		0.15 ± 0.08 (0.06–0.37)	0.21 ± 0.14 (0.05–0.48)	0.16 ± 0.13 (0.05–0.25)
pDC	/μL	10.64 ± 5.96 (3.43–28.11)	12.46 ± 6.17 (5.41–22.23)	9.11 ± 5.14 (4.5–13.7)
mDC ^e^		0.26 ± 0.14 (0.098–0.63)	0.29 ± 0.13 (0.095–0.41)	0.22 ± 0.07 (0.13–0.33)
mDC	/μL	18.82 ± 8.89 (6.3–38.32)	19.01 ± 10.99 (7.3–45.6)	14.98 ± 4.48 (8.78–23.17)

Table legend: CM: central memory; DCs: dendritic cells; EM: effector memory; mDCs: myeloid dendritic cells; pDCs: plasmacytoid dendritic cells; RTE: recent thymic emigrants; TEMRA: terminally differentiated; TfH: T follicular helper cells; Tregs: regulatory T cells. ^a^ % of CD4+ T lymphocytes, ^b^ % of CD8+ T lymphocytes, ^c^ % of CD4+CD45RO+ T lymphocytes, ^d^ % CD19+ B lymphocytes, ^e^ % WBC. Values are expressed as mean ± standard deviation. The ranges (minimum and maximum values) are indicated in brackets.

## Data Availability

The data are available from the corresponding author on reasonable request.

## Supplementary Materials

The following supporting information can be downloaded at https://www.mdpi.com/article/10.3390/jcm12247579/s1, Table S1: Monoclonal antibodies used for extended immunophenotype; Figure S1: Gating strategy to identify lymphocytes; Figure S2: Gating strategy for conventional lymphocyte populations; Figure S3: Gating strategy for CD4 and CD8 T cell subpopulations in a patient affected by 22q11.2 deletion syndrome.
