# Supplementary material for: Distinct Immunophenotypic Features in Patients Affected by 22q11.2 Deletion Syndrome with Immune Dysregulation and Infectious Phenotype
Source: J Clin Med. 2023 Dec 8;12(24):7579. doi: 10.3390/jcm12247579 (PMC10743584; doi:10.3390/jcm12247579)

Detail of the panels and gating strategies.

Fresh whole blood was processed within 5 hours after collection; it was split into 6 different panel fractions to discriminate specific cell populations and to assess:

1) General lymphocyte populations;
2) CD4 and CD8 T-cell subpopulations;
3) Regulatory T cells;
4) Follicular T helper cells;
5) B-cell subpopulations;
6) Dendritic cell subsets.

Monoclonal antibodies used for extended immunophenotype are described in Table S1. In each panel a forward scatter height (FSC-H) versus forward scatter area (FSC-A) density plot was used to exclude doublets, FSC versus side scatter (SSC) gating was used to identify white blood cells and to exclude debris; lastly, gating by CD45 vs SSC we identified cells of interest (Figure 1).

1) B cells (CD19+), T cells (CD3+), and Natural Killer (NK; CD16+/CD56+) cells were separated from the lymphocyte collection gate using characteristic markers, as shown in Figure S2. Moreover, the CD4 versus CD8 dot plot allowed the discrimination of four different cell types: CD4+ and CD8+ T cells, double-negative CD4-CD8- and double-positive (CD4+CD8+) T cells may also be identified.
2) Originating from the lymphocyte gate, T cells (CD3+), CD4+ and CD8+ T cells were separated and further divided into naïve (CD45RA+CD62L+), central memory (CD45RA−CD62L+), effector memory (CD45RA−CD62L−), terminal effector cells (CD45RA+CD62L−) and recent thymic emigrants (RTE: CD45RA+CD62L+CD31+). Gating strategy for CD4 and CD8 T-cell subpopulations is shown in Figure S3.
3) Circulating regulatory T cells (Treg) were identified as a CD4+CD25+/++CD127low/− cell population. T helper (CD3+CD4+) cells were separated based on the lymphocyte collection gate. The gating strategy of CD25 versus CD127 plot allowed the identification of regulatory T cell population. Additionally, the expression of CD45RA was evaluated to estimate the amount of naïve Treg cells.
4) The expression of CD185 (CXCR5) on memory CD4+ T lymphocytes allowed the identification of T follicular helper (TfH) cells. T helper (CD3+CD4+) cells were separated based on the lymphocyte collection gate. CD45RO+ identified memory T helper cells and the expression of CD185 on CD3+CD4+CD45RO+ was determined.
5) B cells (CD19+) were separated from the lymphocyte gate. The combination plot of CD27 and IgD allowed the identification of naive (CD27-IgD+) and memory (CD27+) subpopulations. Based on CD27+ gate, unswitched and switched memory B cells were detected by gating IgD versus IgM (unswitched memory: CD19+CD27+IgM+IgD+; switched memory: CD19+CD27+IgM-IgD-). Transitional B cells were identified by CD38+++ and IgM+++ coexpression; in the same dot blot plasmablasts were identified based on high expression of CD38 and absence of IgM. Moreover the CD21 versus CD38 dot allowed the discrimination of CD21lowCD38low B cell population.

6) Dendritic cell gating strategy was set as previously described (Orsini G, Legitimo A, Failli A, Massei F, Biver P, Consolini R. Enumeration of human peripheral blood dendritic cells throughout the life. Int Immunol. 2012 Jun;24(6):347-56. doi: 10.1093/intimm/dxs006 for DCs;): because PBDCs are characterized by side scatter (SS) and forward scatter (FS) parameters similar to peripheral blood mononuclear cells (PBMCs), the acquisition gate was made on the basis of SS and FS characteristics of lymphocytes and monocytes, excluding granulocytes and debris; as DCs are rare events, fifty thousand events were acquired for each sample. These events were analyzed in a dot plot of side scatter versus CD33, for the myeloid DC subset, or CD123 for plasmacytoid one, to identify the CD33+ (or CD123+) cells. These cells were examined for the expression of CD85k, CD14 and CD16 in an appropriate dot plot; cells resulted positive for the antigen CD85k and negative, or at a low expression, for CD14 and CD16 were selected. Then, a further dot plot of CD85k versus CD33 (or CD123) was created to display mDCs or pDCs in a characteristic round shape.

Table S1 Monoclonal antibodies used for extended immunophenotype

Panel	Antigen	Fluorochrome	Company
1) General lymphocyte populations	CD16	FITC	BD Bioscences
	CD56	FITC	BD Bioscences
	CD3	PE	BD Bioscences
	CD45	PerCP-Cy5.5	BD Bioscences
	CD4	PE-Cy7	BD Bioscences
	CD19	APC	BD Bioscences
	CD8	APC-Cy7	BD Bioscences
2) CD4 and CD8 T-cell subpopulations	CD45RA	FITC	BD Bioscences
	CD31	PE	BD Bioscences
	CD3	PerCP	BD Bioscences
	CD4	PE-Cy7	BD Bioscences
	CD62L	APC	BD Bioscences
	CD45	APC-Cy7	BD Bioscences
	CD8	V450	BD Horizon
3) Regulatory T cells	CD45RA	FITC	BD Bioscences
	CD4	PE	BD Bioscences
	CD3	PerCP	BD Bioscences
	CD25	PE-Cy7	BD Bioscences
	CD127	Alexa Fluor	BD Pharmingen
	CD8	APC-Cy7	BD Bioscences
4) Follicular T helper cells	CD185 (CXCR5)	BB 515	BD Horizon
	PD1	PE	BD Bioscences
	CD3	PerCP	BD Bioscences
	CD4	PE-Cy7	BD Bioscences
	CD45RO	APC	BD Bioscences
	CD45	APC-Cy7	BD Bioscences
	CD8	V450	BD Horizon
5) B-cell subpopulations	IgD	FITC	BD Bioscences
	CD24	FITC	BD Bioscences
	IgM	PE	BD Bioscences
	CD21	PE	BD Bioscences
	CD45	PerCP-Cy5.5	BD Bioscences
	CD19	PE-Cy7	BD Bioscences
	CD38	PE-Cy7	BD Bioscences
	CD27	APC	BD Bioscences
	CD38	APC	BD Bioscences
	CD19	APC-Cy7	BD Bioscences
6) Dendritic cell subsets	CD14	FITC	Beckman Coulter
	CD16	FITC	Beckman Coulter
	CD85K	PE	Beckman Coulter
	CD33	PC5	Beckman Coulter
	CD123	PC5	Beckman Coulter
	CD45	APC-Cy7	BD Bioscences

Figure S1: Gating strategy to identify lymphocytes

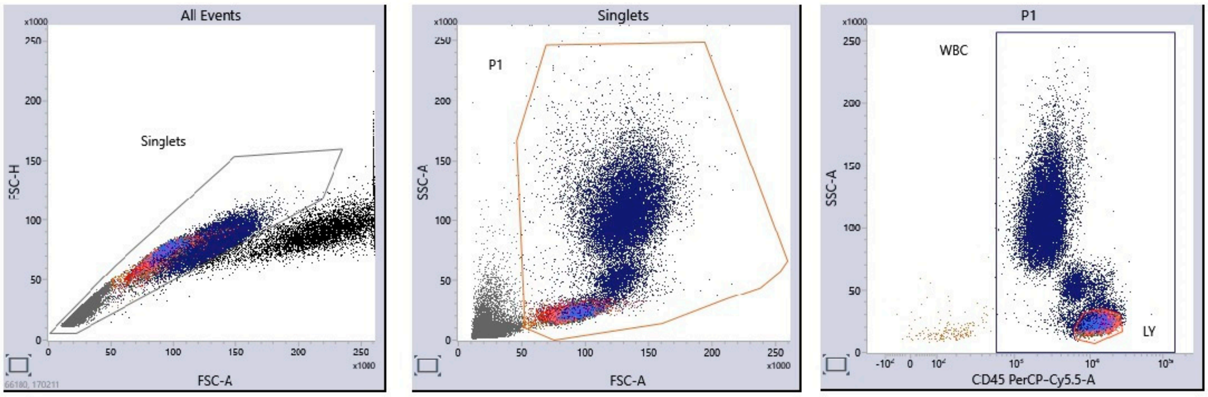

Figure S2: Gating strategy for conventional lymphocyte populations

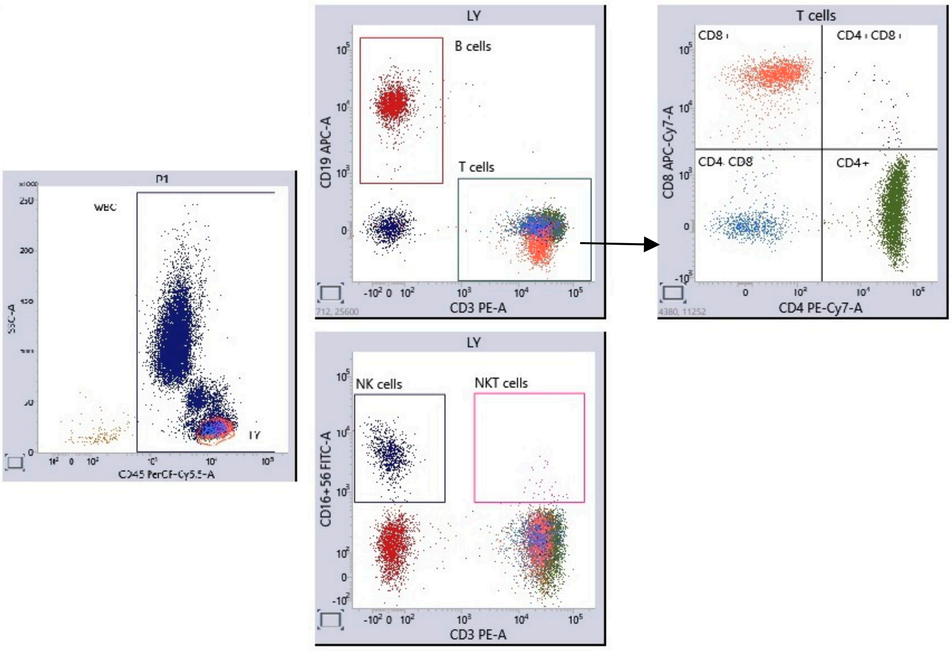

Figure S3: Gating strategy for CD4 and CD8 T-cell subpopulations in a patient affected by 22q11.2 deletion syndrome

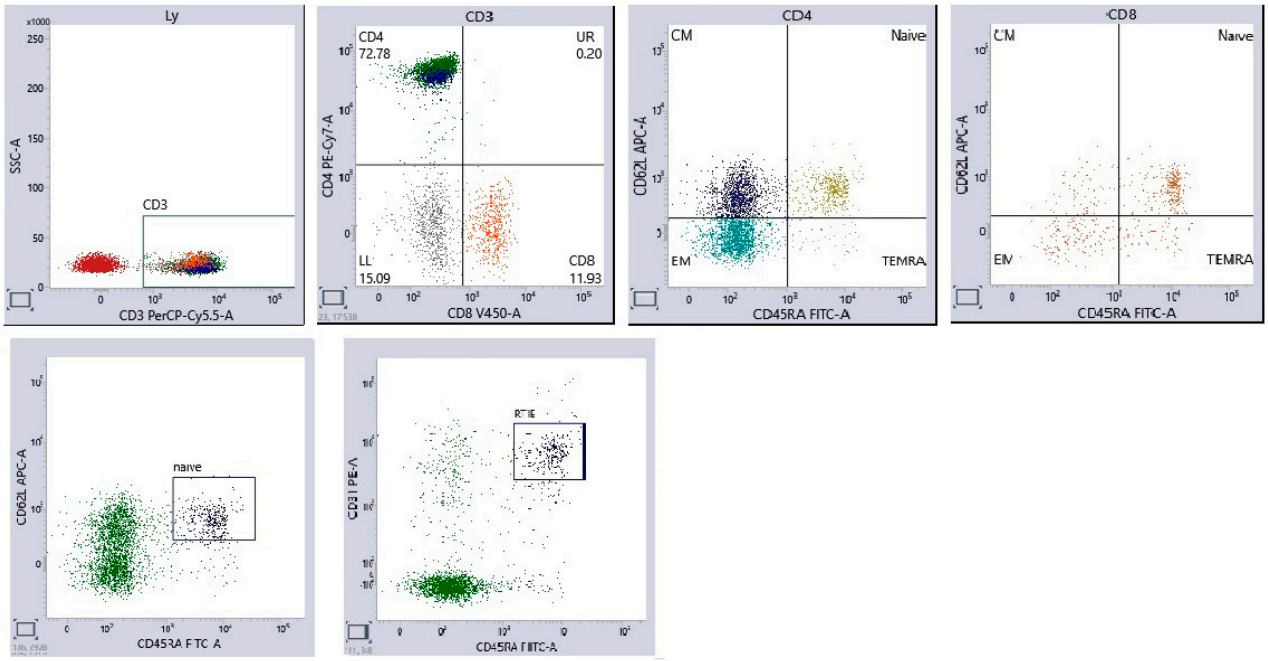

Supplement: Supplementary file 1 [file jcm-12-07579-s001.zip › jcm-2721715-supplementary.pdf]
